# Dualism and its place in a philosophical structure for psychiatry

**DOI:** 10.1007/s11019-018-9841-2

**Published:** 2018-05-19

**Authors:** Hane Htut Maung

**Affiliations:** Department of Philosophy, School of Social Sciences, University of Manchester, Manchester, M13 9PL UK

**Keywords:** Dualism, Psychiatry, Consciousness, Mind–body problem, Philosophy of mind

## Abstract

It is often claimed in parts of the psychiatric literature that neuroscientific research into the biological basis of mental disorder undermines dualism in the philosophy of mind. This paper shows that such a claim does not apply to all forms of dualism. Focusing on Kenneth Kendler’s discussion of the mind–body problem in biological psychiatry, I argue that such criticism of dualism often conflates the psychological and phenomenal concepts of the mental. Moreover, it fails to acknowledge that there are different varieties of dualism, and so overlooks the important metaphysical insights of contemporary dualist philosophers. I argue that while the neuroscientific research underpinning biological psychiatry challenges the traditional dualism of René Descartes, it does not pose any problem for the more modern dualism of David Chalmers. It is possible to take seriously the scientific claims of biological psychiatry while holding that this latter form of dualism is true. This has implications for the positioning of the mind–body problem in psychiatry. While the “easy” problem of explaining psychological processes is relevant to the aims of biological psychiatry, psychiatrists need not worry about the “hard” problem of consciousness.

## Introduction

Biological psychiatry is an approach to psychiatry that seeks to explain and conceptualise mental disorders as biological processes in the brain. According to biological psychiatry, mental disorders are viewed as “relatively stable prototypical, dysfunctional patterns of experience and behavior that can be explained by dysfunctional neural systems at various levels” (Walter 2013, p. 2). This is considered to receive support from recent innovations in functional neuroimaging, as well as advances in our understanding of psychiatric genetics, neurophysiology, and cognitive psychology. While not without its critics, such as those who comment on the absence of biomarkers in psychiatry (Bentall 2009; Moncrieff 2010) and those who argue that it neglects the important role of the social environment in constituting mental disorder (Fuchs 2012; Levy 2013; Davies 2016; Cooper 2017), biological psychiatry remains a dominant paradigm in contemporary psychiatric research and practice.

The advances in neuroscientific research that underpin biological psychiatry have altered the ways in which we think about mental disorder and the mind more generally. It is sometimes assumed that this neuroscientific research undermines dualism in the philosophy of mind, which proposes that the mental is ontologically distinct from the physical. In parts of the biological psychiatry literature, it is commonly contended that there is no need to appeal to an immaterial mind to explain mental disorders, because they can be explained by biological processes in the brain. However, as we shall see, dualist positions differ with respect to how the relation between the physical and the mental is characterised. In this paper, I argue that critical discussions of dualism in parts of the biological psychiatry literature fail to appreciate this varied metaphysical landscape and do not apply to all forms of dualism. Such oversight is unfortunate, because it misleadingly conflates different aspects of the mind–body problem and neglects to do justice to important contributions to philosophy of mind by contemporary dualist philosophers. As noted by the philosopher Rachel Cooper, “[w]ithin the psychiatric literature dualism is sometimes presented as being a dead theory” (Cooper 2007, p. 106). Cooper argues that this portrayal of dualism is mistaken and I agree. Ultimately, I show that a certain sort of dualism between the physical and the phenomenal is entirely compatible with a biological approach to psychiatry.

The rest of this paper is in four parts. The first part presents the criticism of dualism found in a subset of the biological psychiatry literature, specifically focusing on Kenneth Kendler’s discussion in “Toward a Philosophical Structure for Psychiatry” (2005). The second part argues that this discussion erroneously conflates two different concepts in the philosophy of mind, namely the psychological and phenomenological concepts of the mental (Chalmers 1996). The third part specifies and articulates two dualist positions, which are the traditional dualism of René Descartes ([1641] 1993) and the modern dualism of David Chalmers (1996). With reference to the causal exclusion problem (Kim 1998), I show that although the former is challenged by biological psychiatry, the latter is not. One can both accept the truth of the latter form of dualism and all the research findings of biological psychiatry. Therefore, the assumption that dualism is incompatible with biological psychiatry is false. The fourth part explores the implications of my analysis for the positioning of the mind–body problem in psychiatry. I suggest that the explanatory gap between the physical and phenomenal, otherwise known as the “hard” problem of consciousness (Chalmers 1995), need not worry psychiatrists, because it does not affect the understanding of the causal processes underlying psychiatric disorders or the development of therapeutic interventions.

Before I proceed further, I offer two clarifications. First, although I am sympathetic to the views of some of the aforementioned critics of the strict biological approach to psychiatry, it is not my intention in this paper to dispute any of the empirical or theoretical claims of biological psychiatry. The target of my criticism is specifically the assumption that these empirical and theoretical claims undermine dualism. Hence, an important constraint I set myself in this paper is to take the science of psychiatry seriously. Second, the main aim of this paper is not so much to advance the debate on the mind–body problem in the philosophy of mind, but rather to utilise key theoretical distinctions in recent philosophy of mind to highlight conceptual errors in some of the claims about the mind–body problem in parts of the biological psychiatry literature. Hence, the paper contributes to the philosophy of psychiatry by promoting greater conceptual clarity in line with current theory in philosophy of mind, clearing up misconceptions about dualism’s compatibility with biological psychiatry, and clarifying which aspects of the mind–body problem are and are not troublesome for the aims of psychiatric research. Accordingly, it is beyond the scope of this paper to review all of the arguments on the mind–body problem in the philosophical literature. Rather, my philosophical defence of dualism in this paper merely consists of showing that a modern version of it is entirely compatible with biological psychiatry.

## Critique of dualism in the psychiatric literature

There has been recent interest in the mind–body problem in the biological psychiatry literature. The psychiatrist Kenneth Kendler offers a particularly detailed discussion of the problem in his paper, “Toward a Philosophical Structure for Psychiatry” (2005). He lays out his vision for a philosophical framework for biological psychiatry, which involves eight desiderata:

1. Psychiatry is irrevocably grounded in mental, first-person experiences. 2. Cartesian substance dualism is false. 3. Epiphenomenalism is false. 4. Both brain → mind and mind → brain causality are real. 5. Psychiatric disorders are etiologically complex, and we can expect no more “spirochete-like” discoveries that will explain their origins in simple terms. 6. Explanatory pluralism is preferable to monistic explanatory approaches, especially biological reductionism. 7. Psychiatry needs to move from a prescientific “battle of paradigms” towards a more mature approach that embraces complexity along with empirically rigorous and pluralistic explanatory models. 8. Finally, we need to accept “patchy reductionism” with the goal of piecemeal integration in trying to explain the complex etiological pathways to psychiatric illness a little bit at a time. (Kendler 2005, p. 433)

These desiderata can be placed into two broad categories. The first four desiderata concern the nature of the relation between the physical and the mental, or the mind–body problem. The last four desiderata concern the sort of explanatory approach that is required to understand psychiatric disorder in biological terms. Kendler’s overall aim is to develop a coherent conceptual framework for psychiatry that permits maximal use of the latest scientific insights into the brain and behaviour.

With respect to the explanatory approach described by the last four desiderata, it is interesting to note that while Kendler supports biological psychiatry, he explicitly eschews explanatory reductionism:

Note that I do not contest that ultimately (in the sense of “weak biology”) all psychiatric illness is biological. What is at issue here is the optimal level in the causal processes underlying psychiatric illness at which intervention can be best focused and understanding most easily achieved. (Kendler 2005, p. 436)

Kendler’s contention is that psychiatric illness is causally complex and cannot be adequately conceptualised at a single level of explanation. Rather, he advocates explanatory pluralism, whereby psychiatric illness is best understood as resulting from the complex interactions of multiple factors from various levels of explanation. Nonetheless, this approach to psychiatry is robustly biological, as it states that these factors and the interactions between them are grounded in biological processes.

With respect to the position on the mind–body problem described by the first four desiderata, Kendler claims that a scientific approach to psychiatry requires a rejection of dualism:

While individual psychiatrists may, for their own personal or religious reasons, continue to advocate mind–body dualism, it is time for the field of psychiatry to declare that Cartesian substance dualism is false. We need to reject definitively the belief that mind and brain reflect two fundamentally different and ultimately incommensurable kinds of “stuff”. Rather, in accord with an overwhelming degree of clinical and scientific evidence, we should conclude that the human first-person world of subjective experience emerges from and is entirely dependent upon brain functioning. (Kendler 2005, p. 434)

Kendler does not give specific details of the “overwhelming degree of clinical and scientific evidence”, but he does make reference to “the growth of neuroscience and molecular biology” offering “rich insights into the basic workings of the human brain” (Kendler 2005, p. 433). The contention is that mental processes, which might formerly have been attributed to the workings of an immaterial mind, can now be explained by neuroscience and molecular biology in terms of physical processes in the brain.

Such scientific insights, according to Kendler, support physicalism, which is the claim that everything in the world, including the mind, metaphysically supervenes on physical features of the world. To clarify the notion of metaphysical supervenience, a set of properties *F* is said to metaphysically supervene on another set of properties *G* if and only if there is no possible world in which there is a difference in *F* without any difference in *G*. Historically, physicalist theories of the mind have appeared in several varieties. The analytical behaviourism associated with the logical positivists (Carnap 1932; Hempel 1949) and to some extent Gilbert Ryle (1949) suggests that mental concepts are completely translatable to behavioural concepts, so that sentences that use such terms as “belief” and “thought” can be replaced by sentences that describe behaviours and dispositions without changing their meanings. This then inspired the type identity theory of Place (1956) and Smart (1959), according to which kinds of mental state are literally identical with respective kinds of brain state. In response to the implausibility of there being such one-to-one correlations between mental state kinds and brain state kinds, functionalism instead suggests that mental states are identified by their respective causal roles relative to sensory inputs, other mental states, and behavioural outputs (Lewis 1966; Armstrong 1968). More revisionarily, eliminative materialism claims that our ordinary notions of mental states are empty and will be eliminated by future neuroscientific findings (Churchland 1981).

Kendler does not explicitly specify the variety of physicalism he is assuming, but his desideratum that “[b]oth brain → mind and mind → brain causality are real” suggests that he does not endorse analytical behaviourism, type identity theory, or eliminative materialism. Rather, he appears to assume psychological realism combined with ontological physicalism. In other words, mental states are real and causally efficacious states, but they nonetheless metaphysically supervene on physical features of the world. What is unequivocal, however, is his contention that “[b]y rejecting dualism, we accept that all psychiatric disorders are biological” (Kendler 2005, p. 434). In other words, because one cannot appeal to the workings of an immaterial mind, one must explain psychiatric disorder in terms of the processes in a biological system.

Of course, Kendler’s particular view is not necessarily representative of the views of all psychiatrists. It is conceivable that there are psychiatrists who are either philosophically inclined towards dualism or who have no strong commitments regarding the metaphysics of the mind–body problem (Moreira-Almeida and Araujo 2015). However, Kendler’s antipathy to dualism is certainly not an uncommon attitude in the research culture of biological psychiatry. As noted in the *Textbook of Biological Psychiatry*:

Most neuroscientifically orientated investigators support a *monistic* view of the mind and brain. … In keeping with the monistic perspective, most brain–mind scientists believe that consciousness is a phenomenon that emerges from the complexity of central nervous system (CNS) development, arising from within the dynamics of the brain, and existing in an embodied and body-centred “subjective space”, totally private to its owner. (Watt and Pincus 2004, pp. 77–78)

Some specific examples of this attitude can be found in the psychiatric literature. In a paper on the persistence of dualism in psychiatric reasoning, Miresco and Kirmayer suggest that “the idea that mind and brain are different entities is no longer credible in medical science” (Miresco and Kirmayer 2006, p. 913). In the context of clinical psychiatric practice, Ventriglio and Bhugra suggest that “abandonment of this faux dualism is the first step in the training of physicians, including psychiatrists” (Ventriglio and Bhugra 2015, p. 370). Interestingly, a survey of over six-hundred psychiatrists by Moreira-Almeida and Araujo (2015) found that over half of the participants initially assumed physicalism regarding the mind, but also found that a significant number altered or refined their views after attaining greater conceptual clarity through attending a philosophical debate on the mind–body problem. And so, Kendler’s particular view may not be shared by all psychiatrists, but his attitude towards dualism’s relation to biological psychiatry does at least reflect a common attitude in prominent parts of biological psychiatry. Nevertheless, while my critique in this paper is selectively centred on Kendler’s particular view, the ontological conclusion that follows from this critique, namely that a modern variety of dualism is compatible with biological psychiatry, is applicable to biological psychiatry in general.

Although dualism is commonly disparaged in some of the biological psychiatry literature, it has recently been defended in the philosophical literature. This has to a large part been motivated by the perceived failure of physicalism to account for the subjective experience of consciousness (Nagel 1974; Kripke 1980; Jackson 1982). In contemporary philosophy of mind, many notable philosophers endorse dualism as a respectable account of the mental (Chalmers 1996; Langsam 2001; Gertler 2008; Nida-Rümelin 2010; Fürst 2011; BonJour 2013). Moreover, the dualism endorsed by many of these philosophers is nontheistic and compatible with a naturalistic view of the world, and so cannot be claimed to be held merely for “personal or religious reasons” (Kendler 2005, p. 434). In philosophy of psychiatry, Rachel Cooper (2007, pp. 104–106) defends dualism by arguing that the neuroscientific research underpinning biological psychiatry at most tells us about the correlations between mental states and processes in the brain. She proposes that this is insufficient to undermine dualism, because one could still hold that the mental is separate from the physical while accepting that there are reliable correlations between the two. In what is to follow, I go beyond this by showing precisely what it is about the mental that escapes the explanatory net of biological psychiatry. I shall argue that there is confusion between different concepts of the mental in some of the biological psychiatry literature, which results in an unfair misrepresentation of dualism.

## Two concepts of the mental

In his seminal book, *The Conscious Mind: In Search of a Fundamental Theory* (1996), the philosopher David Chalmers notes that mental terms are ambiguous and can be used to express different concepts. He distinguishes two different concepts of the mental, which are respectively the psychological concept and the phenomenal concept (Chalmers 1996, pp. 11–22). The psychological, or functional, concept of the mental is that which concerns the causal processes involved in the production and regulation of behaviour, where behaviour broadly includes perceptual judgements, involuntary responses, voluntary actions, and speech acts. This is the concept of the mental that is of interest to cognitive science. What matters about a mental state according to the psychological concept is its role in the causation and explanation of behaviour.

The phenomenal concept of the mental is that which concerns the subjective quality of experience. When one is involved in perceiving, thinking, and acting, there occur various complex causal processes in one’s brain. However, these processes do not usually go on “in the dark”, but are associated with a phenomenal feel (Chalmers 1996, p. 4). They are accompanied by a first-person subject of experience, that is to say, by consciousness. To borrow a phrase from Thomas Nagel (1974), there is usually “something it is like” to be in a given mental state.

These two concepts of the mental are often conflated in ordinary language. For example, “pain” can be taken to mean a kind of psychological state that normally results from actual or potential tissue damage and that normally produces aversive reactions, verbal reports such as “it hurts” and “I am in pain”, increased sympathetic nervous system activity, and so on. However, it can also be taken to mean the phenomenal quality that normally accompanies this psychological state. One reason why these two concepts are often conflated is that the two tend to co-occur (Chalmers 1996, p. 17). For example, the psychological state of pain that results from tissue damage and produces aversion is normally accompanied by the phenomenal feel of pain. Nonetheless, while they do indeed tend to occur together, it is important to acknowledge the conceptual distinction between the two. We apply the psychological concept of the mental when we are interested in the causation and explanation of behaviour, but we apply the phenomenal concept of the mental when we are interested in the subjective quality of experience.

An unfortunate consequence of such conflation between the two concepts of the mental is that it can lead to confusion in the discussion of the mind–body problem, often in such a way that unfairly trivialises the phenomenal concept. As noted by Chalmers, it is often the case that a discussion of consciousness “will start by investing the problem with all the gravity of the problem of phenomenal consciousness, but will end by giving an explanation of some aspect of psychological consciousness, such as the ability to introspect” (Chalmers 1996, p. 26). By equivocating between the two concepts, the phenomenal is misleadingly defined away in terms of the psychological without being properly addressed.

I argue that Kendler (2005) is guilty of such equivocation in his aforementioned paper on the mind–body problem in biological psychiatry. He begins with the claim that psychiatry is irrevocably grounded in first-person subjective experience:

Our central goal as a medical discipline is the alleviation of the human suffering that results from dysfunctional alterations in certain domains of first-person, subjective experience, such as mood, perception, and cognition. Our nosological constructs are largely composed of descriptions of first-person experiences (e.g., sad mood, hallucinations, and irrational fears). The clinical work of psychiatry constantly requires us to assess and interpret the first-person reports of our patients. Many of the target symptoms that we treat can only be evaluated by asking our patients about their subjective experiences. (Kendler 2005, p. 433)

The emphasis on “first-person, subjective experience” in this passage strongly suggests that Kendler is writing about the phenomenal concept of the mental. However, he then lists “mood, perception, and cognition” as certain domains of this subjective experience. While mood certainly has a phenomenal aspect, cognition is very much a psychological concept which refers to the causal dynamics of information processing leading to the production of behaviour.

Later in the paper, Kendler argues that “mental processes carry critical causal information about human behavior” (Kendler 2005, p. 434). This suggests that he is applying the psychological concept of the mental, as he is characterising mental processes in terms of their causal roles. However, in the same paragraph, he runs the psychological and the phenomenal concepts together with the claim that “subjective, first-person mental phenomena have causal efficacy in the world” (Kendler 2005, p. 434). I argue that this is claim is misguided, because it fudges the conceptual distinction between the two. While it may indeed be the case that a mental state with a particular causal role is associated with a particular subjective quality of experience, it is possible to keep the two concepts apart. Hence, when Kendler mentions the role of humiliation in the aetiology of psychiatric disorder but then states that “[h]umiliation and loss are classical, subjective, first-person experiences” (Kendler 2005, p. 436), he is conflating two concepts of “humiliation”. Like the aforementioned example of “pain”, the term “humiliation” can be taken to mean a subjective experience with a particular phenomenal quality, but it can also be taken to mean a psychological state characterised by its causal role in producing a variety of behaviour in response to a certain sort of social situation. Again, while these tend to co-occur, they can at least be kept apart conceptually. One can characterise the causal role of a mental process without having to invoke its associated phenomenal quality and vice versa.

As noted by Chalmers (1996, p. 23), such conflation does not usually result in too many problems in everyday discourse, because the two concepts usually co-occur. For philosophical purposes, however, this conflation is a serious issue. Equivocating between the two concepts of the mental leads to misunderstanding about the phenomenon of subjective experience and to confusion about what aspect of the mind–body problem is relevant to psychiatry. As I shall show in more detail later, this can have unfortunate implications for psychiatric research and practice, because it can result in researchers talking cross purposes and generate confusion regarding the epistemic standard for psychiatric explanation. In the next section, I examine how the distinction between the two concepts of the mental relates specifically to the compatibility of dualism and biological psychiatry.

## Varieties of dualism

As well as distinguishing the psychological and phenomenal concepts of the mental, it is important to recognise different varieties of dualism. These are the traditional dualism proposed by René Descartes in his *Meditations on First Philosophy* ([1641] 1993) and the modern dualism proposed by David Chalmers in *The Conscious Mind: In Search of a Fundamental Theory* (1996), which I henceforth respectively call Descartes-style dualism and Chalmers-style dualism. In what is to follow in this section, I describe these varieties of dualism in turn, emphasise the key differences between them, and show why in light of these differences the latter but not the former variety of dualism is wholly compatible with the scientific claims of biological psychiatry.

Descartes-style dualism proposes a metaphysical distinction between two kinds of substance, namely the physical *res extensa* and the mental *res cogitans*. The *res extensa* is spatially extended, divisible, and proceeds mechanistically in accordance with laws. By contrast, the *res cogitans* is the thinking being which is unextended and indivisible:

… on the one hand I have a clear and distinct idea of myself, insofar as I am merely a thinking thing and not an extended thing, and because on the other hand I have a distinct idea of a body, insofar as it is merely an extended thing and not a thinking thing, it is certain that I am really distinct from my body, and can exist without it. (Descartes [1641] 1993, p. 51)

While Descartes states that the *res extensa* and the *res cogitans* are metaphysically distinct, he nonetheless suggests that they causally interact with each other. Regarding the mental influencing the physical, he proposes that “when we will to walk or move our body in some other manner, this volition makes the gland drive the spirits toward the muscles conducive to this effect” (Descartes [1649] 1989, p. 42). Conversely, regarding the physical influencing the mental, he suggests that “if we see some animal coming toward us, … images in the brain compose only a single [image] of it on the gland, which, acting immediately on the soul, makes it see the animal’s shape” (Descartes [1649] 1989, p. 38). Hence, Descartes-style dualism posits causal interactions between two metaphysically distinct kinds of substance. Other advocates of this variety of dualism have included Popper and Eccles (1977), W. D. Hart (1988), and John Foster (1991).

Chalmers-style dualism also accepts a metaphysical distinction between the physical and the mental, but focuses specifically on the phenomenal concept of the mental. In other words, it states only that consciousness is a fundamental feature which is ontologically separate from the physical features of the world. Apart from Chalmers (1996), this variety of dualism is advocated by several contemporary philosophers, including Brie Gertler (2008), Martine Nida-Rümelin (2010), Martina Fürst (2011), and Laurence BonJour (2013). As I alluded to earlier, Chalmers-style dualism is inspired by the arguments of Thomas Nagel (1974), Saul Kripke (1980), and Frank Jackson (1982), who show the failure of physicalism to account for the subjective quality of experience by revealing epistemic and modal gaps between the physical and phenomenal. Physical information is ultimately cast in terms of structure and dynamics, but this third-person structural and dynamical information does not entail the presence of first-person subjective experience. For example, one could provide a physical account of what happens when a system perceives a certain stimulus, including information about the structure and dynamics of the stimulus, the physics of signal transduction, the mechanisms of subsequent processing, and the resulting changes in the configuration of the system, but whether or not these processes are accompanied by a phenomenal “something it is like” remains an open question. Chalmers argues that this is the case for any neuroscientific account of the mind:

Like cognitive models, these have much to offer in explaining psychological phenomena, such as the varieties of awareness. They can also tell us something about the brain processes that are correlated with consciousness. But none of these accounts explains the correlation: we are not told why brain processes should give rise to experience at all. … At best, a neurophysiological account might be able to explain why the relevant psychological property is instantiated. The question of why the relevant property in question should be accompanied by conscious experience is left unanswered. (Chalmers 1996, p. 115)

The crux is that no matter how sophisticated a physical account may be, facts about structure and dynamics can only yield further facts about structure and dynamics, but the existence of consciousness fundamentally remains an extra fact beyond these physical facts (Chalmers 1996, pp. 121–122). Therefore, physicalism is false and dualism is true. Consciousness must be accepted as being an ontologically fundamental “extra ingredient” (Chalmers 1995).

There are important similarities and differences between the aforementioned varieties of dualism. Both varieties of dualism entail a metaphysical distinction between the physical and the phenomenal, and so state that the phenomenal is not metaphysically supervenient on the physical. However, Chalmers-style dualism posits that physical events and phenomenal events are correlated via psychophysical laws, and so concedes that phenomenal properties supervene nomologically on physical properties (Chalmers 1996, p. 87). That is to say, given a set of psychophysical laws, certain physical properties are associated with certain phenomenal properties in the possible worlds where those laws hold. By contrast, Descartes-style dualism allows for causal interactions between the physical and the mental, but denies such a relation of nomological supervenience. Hence, it asserts a much less robust relation between the physical and the phenomenal.

A further difference between the two varieties of dualism concerns the specification of the phenomenal concept of the mental. As noted above, both varieties of dualism effectively entail a metaphysical distinction between the physical and the phenomenal. However, only Chalmers-style dualism explicitly distinguishes the psychological and phenomenal concepts of the mental. By contrast, Descartes-style dualism tends to run the different concepts of the mental together. Indeed, Chalmers notes that Descartes “assumed that everything psychological that is worthy of being called mental has a conscious aspect” (Chalmers 1996, p. 12). Hence, while Descartes-style dualism entails a metaphysical distinction between the physical and phenomenal, its running together of the psychological and phenomenal concepts of the mental suggests that it also amounts to a dualism between the physical and the psychological. This is further supported by Descartes’ characterisation of the mind as a “thinking thing” that is involved in the causation of behaviour, which is suggestive of the psychological concept of the mental.

Having discerned the different varieties of dualism, I now show that Kendler’s (2005) blanket criticism of dualism promises more than it can deliver. Only Descartes-style dualism, but not Chalmers-style dualism, is challenged by the scientific claims of biological psychiatry. Hence, if one accepts biological psychiatry, then one can still hold that Chalmers-style dualism is true.

This can be understood by appealing to Jaegwon Kim’s (1998) causal exclusion problem, according to which if *C* is causally sufficient for the occurrence of *E*, then *E* is not caused by any other property distinct from *C*. Hence, if a brain state is causally sufficient for the occurrence of a certain behaviour, then this behaviour cannot be caused by anything distinct from this brain state. List and Stoljar offer the following formulation of the problem:

(1) Being in pain causes me to wince.

(2) Being in phys causes me to wince (where ‘phys’ denotes some overall physical state that I am in).

(3) Being in pain is distinct from being in phys.

(4) If being in phys causes me to wince, nothing distinct from being in phys causes me to wince. (List and Stoljar 2017, p. 96)

Here, (1) and (2) are empirical claims, while (4) is a principle of causation, commonly called the exclusion principle. The reasoning is supposed to go from the acceptance of (1), (2), and (4) to the rejection of (3).

Of course, the causal exclusion problem is not uncontested and some philosophers reject it (Bennett 2003; Loewer 2007). If one rejects the exclusion principle, then one can still hold that Descartes-style dualism is true. However, if one does assume the exclusion principle, then it becomes clear why Descartes-style dualism is undermined by biological psychiatry. Descartes-style dualism and biological psychiatry offer competing explanations for the causation of behaviour. As noted earlier, Descartes-style dualism posits that the immaterial mind causally interacts with the material body to produce behaviour, hence satisfying premise (1) in List and Stoljar’s formulation. However, the neuroscience underpinning biological psychiatry suggests that a physical brain state is causally sufficient to cause such behaviour, hence satisfying (2). According to the exclusion principle (4), if a physical brain state is causally sufficient for the behaviour, then the behaviour is not caused by anything distinct from the physical brain state. Therefore, if the claims of biological psychiatry are accepted, then the *res cogitans* of Descartes becomes explanatorily redundant. With respect to the causation of behaviour, there is no room for an immaterial mind that is distinct from the brain.

This problem does not apply to Chalmers-style dualism. With respect to the physical world, Chalmers concedes that “[t]he best evidence of contemporary science tells us that the physical world is more or less causally closed: for every physical event, there is a sufficient physical cause” (Chalmers 1996, p. 125). This satisfies premise (2) of List and Stoljar’s formulation of the causal exclusion problem. However, whether or not (1) is satisfied can remain an open question for the Chalmers-style dualist. Indeed, Chalmers is happy to contend that “the question of the causal relevance of experience remains open, and a more detailed theory of both causation and of experience will be required before the issue can be settled” (Chalmers 1996, p. 160). In what is to follow, I argue that Chalmers-style dualism avoids the causal exclusion problem and remains compatible with biological psychiatry regardless of whether or not a causal role is granted to experience.

If the causal relevance of experience is denied and (1) is not satisfied, then this would lead to a form of epiphenomenalistic dualism. This would avoid the causal exclusion problem, because while it accepts that the phenomenal is metaphysically distinct from the physical, it only grants the latter a role in the causation of behaviour. This is compatible with the claims of biological psychiatry, because it does not dispute the causal story of how psychiatric disorders are physically produced by biological processes. However, this is not Chalmers’ preferred option. While he is open to the idea that experience might be causally irrelevant, he explicitly states that “I do not describe my view as epiphenomenalism” (Chalmers 1996: p. 60).

The other option is to accept the causal relevance of experience and hence satisfy (1). Nonetheless, as List and Stoljar (2017) argue, Chalmers-style dualism can avoid the causal exclusion problem even if (1) is satisfied. The reason concerns the ambiguity of the term “distinct” in premises (3) and (4). Recall the exclusion principle (4), which suggests that if a brain state is causally sufficient for the occurrence of a certain behaviour, then this behaviour cannot be caused by anything distinct from this brain state. Dualism (3) states that phenomenal consciousness is distinct from the physical brain state. List and Stoljar note that “distinct” is ambiguous and can mean different things, including metaphysically distinct and nomologically distinct. Indeed, all varieties of dualism state that the phenomenal is numerically and metaphysically distinct from the physical. However, Chalmers-style dualism, insofar as it posits psychophysical laws between the phenomenal and the physical, concedes that they are not nomologically distinct. That is to say, while they are numerically and metaphysically distinct, they are not nomologically distinct because there is a robust psychophysical relation between them, as discussed earlier. This ambiguity can be exploited to overcome the causal exclusion problem. The Chalmers-style dualist can concede what List and Stoljar (2017, pp. 103–104) take to be the only defensible version of (4), which states that if a brain state is causally sufficient for the occurrence of a certain behaviour then this behaviour cannot be caused by anything nomologically distinct from the brain state, while also accepting a version of (3) which states that the phenomenal is numerically and metaphysically distinct from, but nomologically related to, the physical.

Importantly, this solution is not available to Descartes-style dualism, which denies such a nomological relation between the physical and the phenomenal. Because such a relation is denied, the Descartes-style dualist cannot exploit the ambiguity between metaphysical distinctness and nomological distinctness to avoid the causal exclusion problem. Therefore, Chalmers-style dualism, but not Descartes-style dualism, is compatible with biological psychiatry, because it can avoid the causal exclusion problem by exploiting the ambiguity inherent in the problem.

What I have shown in this section is that some of the literature in biological psychiatry conflates different varieties of dualism, but the critique offered in the literature is only applicable to Descartes-style dualism. Chalmers-style dualism, however, is entirely compatible with biological psychiatry and indeed with science in general:

Further, nothing about this view contradicts anything in physical theory: rather, it supplements that theory. … To capture the spirit of the view I advocate, I call it naturalistic dualism. It is naturalistic because it posits that everything is a consequence of a network of basic properties and laws, and because it is compatible with all the results of contemporary science. (Chalmers 1996, p. 128)

Hence, one can that accept that this sort of dualism is true while taking seriously the claim that psychological processes, including psychiatric disorders, are grounded in biological processes in the brain. In the following section, I explore what implications this has for the positioning of the mind–body problem in psychiatry.

## Positioning the mind–body problem in psychiatry

The aforementioned distinctions between two concepts of the mental and two varieties of dualism show that the mind–body problem is not monolithic, but can be factored into different parts. Chalmers (1995) factors it into an “easy” problem and a “hard” problem. The “easy” problem is the problem of explaining the psychological processes involved in the causation of behaviour in terms of physical processes in the brain. The “hard” problem concerns the question of why these physical processes are accompanied by the phenomenal quality of experience, instead of going on “in the dark” (Chalmers 1996, p. 4).

It should be noted that calling the problem of explaining psychological processes the “easy” problem is not intended to minimise the challenges associated with the task. Here, “easy” and “hard” are to be taken as relative terms. Chalmers concedes that locating the brain mechanisms underlying psychological processes is an extremely difficult project and that modern neuroscience is far from achieving a complete explanation of such processes. However, while this project is empirically and technically challenging, it does not pose such a deep metaphysical mystery. The details may currently be missing, but it is at least intelligible how psychological processes involved in the causation of behaviour could eventually be explained in terms of the structure and dynamics of a physical system. However, the problem of why such properties should be accompanied by a phenomenal quality of experience remains a “hard” metaphysical puzzle.

In some parts of the biological psychiatry literature, it is supposed that the “hard” problem is what has made neuroscientific research into psychiatric disorder so challenging. The neuroscientist Jonathan Roiser makes such a claim in his recent article in *The Psychologist*:

This disconnect between modern neuroscience research and mental health practice partly reflects the unresolved ‘hard’ problem of consciousness: How does the brain generate experience? … Good science (including clinical science) requires reliable measurement, and neuroscience deals with what can be measured objectively at the level of the brain. … By contrast, clinical characteristic of mental health problems, whether conceptualised as categorical disorders or lying on a spectrum, are based on symptoms that in many cases only exist subjectively. (Roiser 2015, pp. 284–285)

This is not an uncommon claim. In their paper on the medical model in psychiatry, Patil and Giordano also suggest that the “hard” problem presents a particular challenge for psychiatry:

In many ways, the question of what constitutes a mental disorder is related to uncertainties about the nature of mental experience, and the underlying relationship(s) of body, brain and mind. … Yet, as neuroscience probes ever deeper into the workings of the brain, it becomes evident that the “mind” remains somewhat enigmatic, and thus, any attempt to link mental events to biology must confront what Chalmers has referred to as the “hard problem” of consciousness. (Patil and Giordano 2010, p. 1)

However, I argue that such characterisation of the “hard” problem is misleading. This is again related to the conflation of two concepts of the mental. A symptom description, such as “depressed mood”, may indeed be associated with a phenomenal quality which is experienced subjectively, but it also picks out a psychological state that produces tearfulness, aversion to activity, and the verbal report of “I feel depressed”. It is this psychological state that is the *explanandum* when one is seeking to characterise depressed mood in biological terms, as the psychological state is what is causally responsible for producing the observed behavioural manifestations and relevant verbal report. Hence, the mapping of self-reported symptoms onto measurable processes at the neuronal level is actually part of the “easy” problem. It is the task of specifying what neuronal mechanisms underlie the psychological states that have causal roles in producing the relevant behavioural syndromes. For this purpose, it is not necessary to answer the metaphysical question of why these neuronal mechanisms are accompanied by phenomenal qualities.

This confusion regarding the different parts of the mind–body problem is not only philosophically unfortunate, but can have unfavourable implications for neuroscientific research relevant to biological psychiatry. As noted by Brian Earp (2012), a lack of conceptual clarity regarding the mind–body problem can hinder communication and result in researchers talking cross purposes. Earp supports this claim further with Marcel Kuijsten’s observation that “[a]t conferences on consciousness, it is often the case that no two speakers seem to be talking about the same subject.” (Kuijsten 2009, p. 2). In turn, I argue that this can encourage an unrealistic view of what epistemic standard must be met for a satisfying biological explanation of mental disorder. Confusing the different parts of the mind–body problem and misconstruing the “hard” problem as a particular challenge for psychiatry could leave the pessimistic impression that an epistemically and clinically satisfying biological understanding of mental disorder is hindered by the problem of the fundamental metaphysics of consciousness.

I argue that this impression is mistaken. While psychiatrists may legitimately worry about the empirical challenges to explaining mental disorders that fall under the “easy” problem, the relevant explanations are not hampered by the metaphysical “hard” problem. This can be qualified further through consideration of the scientific and clinical aims of biological psychiatry. Henrik Walter characterises biological psychiatry as being focused on “a research-inspired, multi-level approach to understand what psychiatric disorders are, what mechanisms underly signs and symptoms and how an understanding of those mechanisms might help in classification, diagnosis, prognosis, and treatment” (Walter 2013, p. 5). Similarly, Derek Bolton states that the main aims of psychiatric science are “*prediction*, refined by *causal explanatory models*, and, on that basis, if cause–effect relationships are sufficiently strong, making *technological applications* including interventions” (Bolton 2012, pp. 6–7). The “easy” problem is particularly relevant to biological psychiatry, because it is concerned with the elucidation of the causal processes underlying psychological states. Such causal information would not only supply biological explanations of behavioural syndromes, but could help inform predictions and locate targets for therapeutic interventions. While the “hard” problem of why the psychological states are associated with phenomenal qualities is philosophically important, it does not pose a particular challenge for the causal explanatory and therapeutic aims of biological psychiatry.

This epistemic standard for explanation in biological psychiatry might be compared to the epistemic standard for explanation in the rest of medicine. For example, consider the task of scientifically explaining the syndrome of angina pectoris. A satisfactory explanation might cite the processes of atherosclerosis, coronary artery obstruction, myocardial ischaemia, stimulation of chemoreceptors and mechanoreceptors, excitation of afferent fibres, excitation of the spinothalamic tract, projection to the medial and lateral thalamus, and activation of several cortical areas (Foreman 1999). Like the previously mentioned symptom of depressed mood, the symptom of angina pectoris is associated with a certain phenomenal quality of experience. Nonetheless, for the purposes of a satisfactory scientific explanation of angina pectoris, it is not considered necessary for one to raise the metaphysical question of why the biological processes involved are accompanied by such a phenomenal quality. The above sort of structural and dynamical explanation would be considered satisfactory for medical purposes. And so, while the “hard” problem is not resolved, this neither hinders the explanatory and therapeutic aims of medicine, nor does it, by parity of reason, hinder the equivalent aims of psychiatry.

The upshot, then, is that only part of the mind–body problem is of particular relevance to the purposes of biological psychiatry. The task of explaining psychiatric disorders in biological terms involves the “easy” problem of specifying the neural mechanisms that underlie the psychological processes responsible for various kinds of behaviour. The “hard” problem of consciousness need not worry psychiatrists interested in explaining and treating psychiatric disorders. For this reason, Chalmers’ proposed solution to the “hard” problem, namely his dualism between the physical and the phenomenal, neither undermines nor is undermined by biological psychiatry.

Before I conclude, I present two potential implications that my analysis in this paper could have for psychiatry as a clinical practice. The first is broadly analogous with the aforementioned implication for psychiatric research. The conflation of different concepts of the mental can result in confusion about precisely what aspects of the mental do and do not need to be explained in order to arrive at epistemically and clinically satisfying explanations of patients’ problems. My analysis shows that it is a mistake to think that the “hard” problem of consciousness is an obstacle for such explanations of mental disorders. Hence, while biologically oriented psychiatrists may legitimately worry about empirical challenges to explaining patients’ problems, they need not worry about the fundamental metaphysics of consciousness.

The second potential implication for clinical practice is that the proposed compatibility of Chalmers-style dualism with biological psychiatry could appeal to those practitioners who worry that a strict biological approach neglects the subjective aspect of illness. For instance, Irene Switankowsky suggests that “[w]ithout interactive dualism, the patient is treated merely as a diseased body, and the treatments administered exclude the subjective features of the illness” (Switankowsky 2000, p. 578). Hence, if the empirical claims of biological psychiatry undermine dualism as Kendler suggests, then this would present a serious concern for Switankowsky. To be clear, I do not necessarily agree with Switankowsky’s conclusion or with the suggestion that ethical considerations follow from fundamental metaphysics in such a direct way. Nonetheless, the analysis I present could appeal to those practitioners who share Switankowsky’s concern. Although biological psychiatry may be in tension with traditional dualism, the modern dualism I endorse provides such practitioners a way of maintaining the irreducibility of subjective experience while accepting the scientific claims of biological psychiatry.

## Conclusion

There is antipathy to dualism in parts of the psychiatric literature. The contribution of this paper has been to use key theoretical distinctions in recent philosophy of mind to show that such antipathy does not give a fair representation of dualism, because it conflates two concepts of the mental and fails to acknowledge that there are different varieties of dualism. This misrepresentation encourages the misconception that dualism is a defunct theory, as it overlooks the important metaphysical contributions of contemporary philosophers who propose that physicalism fails to account for consciousness and that dualism is the only acceptable account (Chalmers 1996; Gertler 2008; Nida-Rümelin 2010; Fürst 2011; BonJour 2013). Focusing on Kendler’s (2005) discussion of the mind–body problem in psychiatry, I have shown that the criticism of dualism found in the psychiatric literature is only applicable to the traditional dualism proposed by Descartes ([1641] 1993), but not to the modern dualism proposed by Chalmers (1996). The latter variety of dualism contends that first-person subjective experience is a further fact that is numerically and metaphysically distinct from the physical facts, but fully accepts that it is nomologically related to physical processes and that the psychological processes involved in the causation of behaviour are explainable in terms of such physical processes in a biological system. This is entirely compatible with the biological psychiatry thesis that psychiatric disorders are grounded in biological processes. Hence, we can accept that this variety of dualism is true while taking seriously the scientific claims of biological psychiatry. This has broader implications for the positioning of the mind–body problem in psychiatric research. Indeed, the problem of specifying the mechanisms involved in psychological processes, or what Chalmers (1995) calls the “easy” problem, is of relevance to research into the causation and treatment of psychiatric disorders. However, once such understanding is attained, psychiatrists need not lose sleep over the philosophical “hard” problem of why these mechanisms are accompanied by phenomenal qualities.
